# Epidemiology of drug driving: protocol from a national Canadian study measuring levels of cannabis, alcohol and other substances in injured drivers

**DOI:** 10.1186/s12889-020-09176-5

**Published:** 2020-07-06

**Authors:** Manal Masud, Herbert Chan, Shannon Erdelyi, Yue Yuan, Jeffrey R. Brubacher

**Affiliations:** Department of Emergency Medicine, Faculty of Medicine, The University of British Columbia, 828 West 10th Avenue, Vancouver, BC V5Z 1M9 Canada

**Keywords:** Motor vehicle collisions, Road safety, Toxicology analysis, Driving under the influence, Impaired driving, Drug use, Cannabis, Methodology

## Abstract

**Background:**

Drug driving is an emerging global road safety problem. As the prevalence of alcohol-impaired driving decreases, and as more jurisdictions decriminalize or legalize cannabis, it is increasingly important for policy makers to have accurate information on the prevalence and pattern of drug driving. Unfortunately, this data is not widely available and the World Health Organization identifies lack of accurate data on the prevalence of drug driving as an important knowledge gap.

**Methods:**

In this paper, we discuss the limitations of current methods of monitoring drug use in drivers. We then present a novel methodology from a multi-centre study that monitors the prevalence and pattern of drug use in injured drivers across Canada. This study uses “left-over” blood taken as part of routine medical care to quantify cannabis and other drugs in non-fatally injured drivers who present to participating emergency departments after a collision. Toxicology testing is done with waiver of consent as we have procedures that prevent results from being linked to any individual. These methods minimize non-response bias and have the advantages of measuring drug concentrations in blood obtained shortly after a collision.

**Discussion:**

Our methods can be applied in other jurisdictions and provide a consistent approach to collect data on drug driving. Consistent methods allow comparison of drug driving prevalence from different regions. Data from this research can be used to inform policies designed to prevent driving under the influence of cannabis and other impairing drugs.

## Background

Impaired driving is a major cause of road traffic injuries and fatalities. Alcohol is the most widely recognized cause of impaired driving. Globally, 17% of all traffic deaths, and millions of traffic injuries, are attributed to alcohol [1]. Cannabis, the second most commonly used impairing drug in the world after alcohol, [2] is another common cause of impaired driving. Several countries, including Canada and several states in the US, have decriminalized recreational cannabis use.. Cannabis legalization has potential benefits including improved product safety, regulation of sales, tax revenue, and an opportunity to eliminate the illicit cannabis market. However, there is concern that legalization may result in more cannabis-related harms, especially traffic collisions [3]. In addition to cannabis and alcohol, drivers use many other impairing substances. Stimulants such as cocaine and amphetamines alter mood, impair judgment and inhibitory control. Sedating medications such as benzodiazepines, antihistamines, antidepressants, and opioids can cause drowsiness, slow reaction time, impair cognitive function and tracking ability. The crash risk with many of these substances, although lower than for alcohol, is as high as or even higher than that associated with cannabis [4–8]. Moreover, in many countries the combined prevalence of driving after using these other impairing substances, alone or in combination, is higher than that for driving after using alcohol or cannabis [9, 10]. As such, the World Health Organization (WHO) considers drug driving as an emerging road safety issue [11]. Policy makers require objective evidence on the prevalence and demographics of drivers using alcohol, cannabis and/or other drugs, especially for drivers involved in collisions. However, this data is not readily available. The 2016 WHO policy brief on drug driving identifies lack of accurate data as an important knowledge gap [5]. Key considerations for collecting this data include the time of measurement, the decision to obtain urine, saliva or blood samples, the choice of driver population and study design.

### Timing of measurement

Many drugs have complex pharmacokinetics that make detection levels obtained hours after a crash difficult to interpret. For example, the main psychoactive compound in cannabis is Δ-9-tetrahydrocannabinol (THC). When cannabis is smoked, blood THC levels peak at > 50 ng/mL within 10 min. In people who use cannabis occasionally, THC is rapidly distributed into body tissue and by 3–4 h after smoking, THC levels are < 2 ng/mL and impairment is usually resolved [12]. Following distribution, levels decline more slowly as THC is metabolized and excreted. After ingesting cannabis edibles, the time to peak THC level is delayed, usually around 4 h, and the impairing effects are prolonged up to 8 h. People who use cannabis frequently may have THC > 2 ng/mL for days after last use due to accumulation of THC in body tissue [13–15]. These factors make it difficult to know if a low THC level measured 3–4 h after a crash corresponds with a high THC level and probable impairment at time of crash in an occasional user, or if it represents a chronic user who consumed cannabis yesterday and was not impaired. Therefore, THC levels are easiest to interpret if they are measured shortly after a collision making it important to obtain blood as soon as possible after a crash. Similarly, many recreational drugs, such as cocaine, amphetamines, and opioids also have complex pharmacokinetics making levels obtained long after an event difficult to interpret [16, 17]. For these drugs, levels are easiest to interpret when samples are obtained as soon as possible after a collision.

### Saliva, urine or blood?

Urine testing is commonly used to screen for psychoactive drug use. Unfortunately, urine tests have little correlation with impairment or recent use because they typically detect inactive metabolites of psychoactive drugs which may have been used days or weeks previously [18]. Oral fluid (saliva) is a promising medium for drug testing in drivers [18–20]. Roadside oral fluid testing devices detect presence or absence of a small panel of common drugs, but do not measure actual concentration and correlate poorly with impairment [21]. Saliva can be also used for broad spectrum drug screens and/or for quantifying drug concentration, but these tests require using advanced laboratory techniques and a larger volume of saliva which cannot be provided by all drivers. A major limitation is that saliva THC concentrations, regardless of how they are measured, correlate poorly with blood levels and/or with impairment [18–20, 22]. Thus, *blood is the preferred body fluid for THC and other drug detection and quantification* as it allows broad spectrum testing and there is a better correlation between drug concentration in blood and pharmacological effect [18, 22–25].

### Driver population and study design

The epidemiology of drug driving in most countries is poorly studied and based on roadside surveys, coroner’s reports, traffic records, self-reported surveys or hospital studies of injured drivers. In roadside surveys, researchers select a sample of drivers at a given time and place and typically obtain a saliva sample which is later analyzed for drugs. Some surveys also ask for blood samples. Roadside surveys sample a large number of drivers over a short time period and provide useful information on drug driving. However, these surveys are limited by high refusal rates and possible selection bias if drivers who consumed drugs are more likely to refuse. Moreover, surveys are often conducted during weekend evenings or nights when alcohol use is higher, so findings may not apply to other days of the week or times of day. As most roadside surveys analyze cannabis and other drugs in oral fluid, findings may not correlate with recent use or impairment as explained earlier. Because of high cost and logistic challenges, roadside surveys are infrequently performed, making them poorly suited for long term monitoring. Coroner’s data often includes toxicology test results from fatally injured drivers and provides a useful indicator of the prevalence of drug driving and types of drugs used. However, coroner’s data can be susceptible to selection bias if drug testing is based on suspicion of drug use and not performed routinely on all drivers. Comparison between jurisdictions is limited because toxicology testing protocols differ between regions and may detect different drugs. If fatally injured drivers survive the crash for a period of time, drug levels will decline with metabolism and post-mortem levels will not reflect levels at time of collision. Interpreting post-mortem drug levels is further complicated by *post-mortem redistribution* which for some drugs (such as cannabis) can result in significantly different results for post-mortem versus pre-mortem drug levels [26–28]. Traffic records (impaired driving citations) are often used to monitor impaired driving. However, in many jurisdictions impaired driving citations do not specify whether impairment was due to alcohol or drugs. Further, police may fail to recognize drivers who consumed drugs. We compared toxicology results from injured drivers to police collision reports and found that police identified only 6% of drivers who tested positive for THC [29]. In addition, the number of impaired driving citations depends on both the prevalence of impaired driving and on enforcement intensity: increased enforcement could result in more citations even if the actual prevalence of drug driving is unchanged. Self-report surveys ask questions about driving after using cannabis or other drugs. Surveys are subject to selection, recall and reporting biases. In addition, many surveys lack precision because they ask about drug use before driving in a given time period (e.g., previous month) instead of before a specific driving episode. The prevalence of drug use in injured drivers has been studied in Europe as part of the Driving Under the Influence of Drugs project as well as in Asia, Australia, and North America [9, 10, 30–32]. In Australia, drug testing is mandatory for all drivers involved in a motor vehicle collision – providing reliable estimates of the prevalence of drug use in crash-involved drivers. Prior studies on the prevalence of drug use in injured drivers usually obtained blood or urine samples specifically for research. In countries without mandatory drug testing, this requires that hospital or research staff identify the injured driver in real time and usually obtain verbal or written consent prior to obtaining blood or urine samples. This process can result in non-respondent bias if drivers who use drugs are less likely to participate than other drivers. For example, almost half of the injured drivers approached in a Hong Kong study refused to participate [30]. In a European study, hospitals in several countries were unable to track refusals or missed eligible drivers [32]. This situation makes it difficult to accurately estimate drug prevalence in injured drivers or compare finding from different countries.

### Objective

We present the protocol for a national Canadian study of drug use in injured drivers. This methodology addresses many of the current limitations to drug driving data collection. If adapted in other jurisdictions, these methods would support international comparisons of the prevalence of drug driving in injured drivers, types of drugs used and demographics of drivers who use drugs prior to a collision.

## Methods

### Study design and setting

**Fig. 1:**
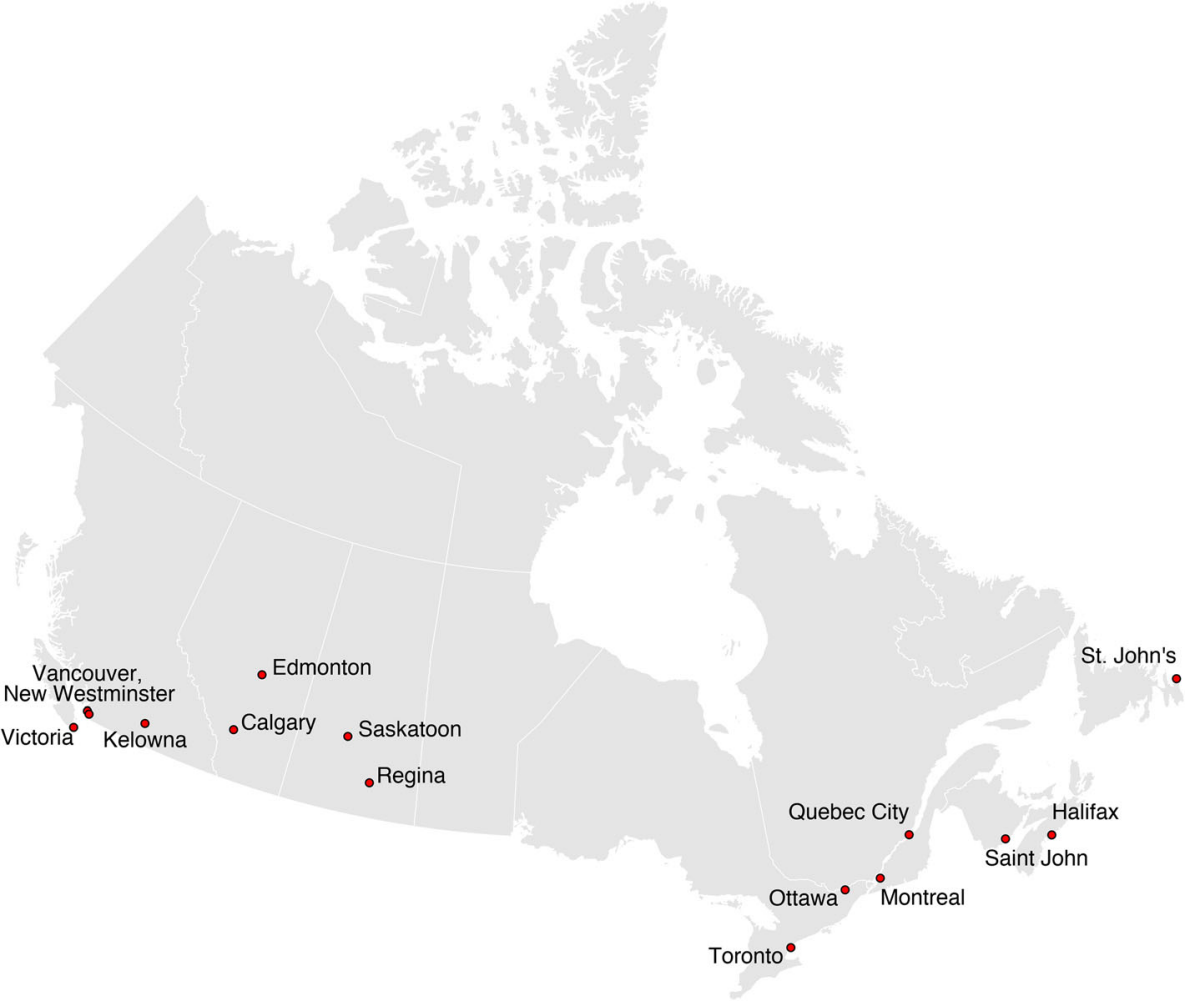
Map of the 15 sites participating in the national drug driving study across Canada*. The study is currently enrolling from 12 of these sites and has approvals in place at the other three. Additional sites may be added pending additional funding and appropriate approvals. *This figure was generated using open access boundary files available from Statistics Canada (Boundary Files, 2016 Census. Statistics Canada Catalogue no. 92–160-X; https://www150.statcan.gc.ca/n1/en/catalogue/92-160-X)

This prospective observational study will obtain data from injured drivers treated in the emergency departments (EDs) in fifteen Canadian cities (Calgary, Edmonton, Halifax, Kelowna, Montreal, New Westminster, Ottawa, Quebec City, Regina, Saskatoon, Saint John, St John’s, Toronto, Vancouver, and Victoria). It is currently enrolling from 12 of these sites and has approvals in place at the other three. (Fig. 1) We plan to enroll 5200 participants over 2 years of recruitment. This number will allow us to report the prevalence of drug driving according to substance (cannabis, impairing medications, etc) disaggregated by injury severity, region, sex, and age group. As this project can be used for public health surveillance to monitor the prevalence of drug driving, we will continue recruitment for more than 2 years if funding allows.

### Eligibility criteria

We include moderately or severely injured drivers of motorized vehicles (e.g. cars, motorcycles, trucks) who visit the ED of a participating hospital and have blood samples obtained within 6 h of the crash. Blood samples are for clinical decision making and are *not* obtained for the purpose of toxicology testing. We exclude drivers of off-road vehicles, cases where no excess blood remains after clinical use and cases that expire in hospital. Injury severity is defined pragmatically as the need to obtain blood for clinical purposes (moderate injury) or need for overnight hospital admission (severe injury). Note that need for hospital admission is used as an indicator of serious traffic injury in Canada, [33] and many other developed countries [34]. Potentially eligible drivers are identified by daily review of ED visit logs and eligibility is confirmed through medical chart review.

### Ethical considerations

**Fig. 2:**
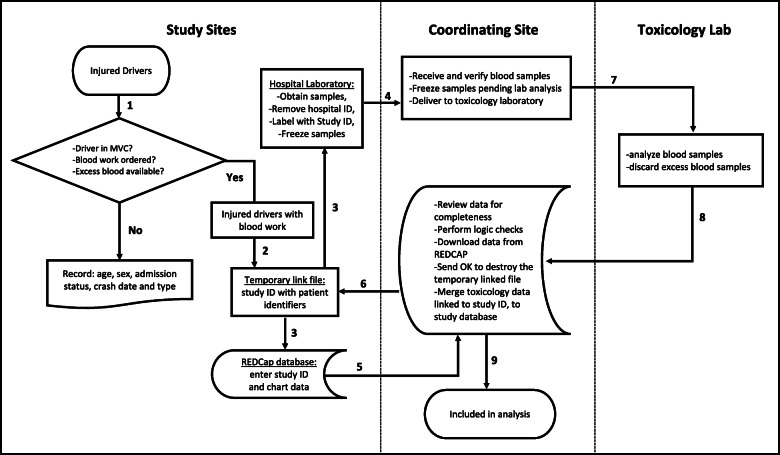
Flow chart of data handling procedures describing the process through which patients are enrolled in the study and data is de-identified. 1 Research assistants review the emergency department (ED) visit logs, identify injured drivers, and determine whether blood work is ordered. 2 Research assistants maintain a temporary link file between patient identifiers and study ID. 3 Chart data linked to study ID is entered into REDCap database. Only basic information is recorded for excluded drivers. The research assistant also provides the laboratory with a list of eligible drivers so that excess blood can be relabelled with study ID and frozen pending shipping to coordinating site. 4 Every 3 months, blood sample are shipped to the coordinating site. At the coordinating centre, blood samples are relabelled with a Toxicology ID replacing the study ID number. A permanent link between study ID number and toxicology ID is maintained at the coordinating site. 5 Once a month, the study coordinator reviews data (in REDCap) for completeness and to conduct error checks. 6 Once data is verified and downloaded, the study coordinator instructs the site research assistants to destroy the temporary link file. After this point there is no linkage between excess blood samples and personal identifiers. 7 The site coordinator sends blood to the toxicology lab for analysis. After blood is analyzed excess samples are destroyed. 8 Toxicology results are sent to study coordinator and linked the to study ID number. The research coordinator merges the results with chart review data. 9 Chart data and toxicology results are stored locally on a password protect computer in a locked office

This study uses “leftover” blood samples that are obtained for clinical purposes and will otherwise be discarded, no extra blood is taken as part of this study. Blood samples are de-identified, and we have procedures in place to prevent linkage between toxicology results and individual drivers (Fig. 2). As a result, we have research ethics board (REB) approval for waiver of consent at all study sites. Waiver of consent greatly strengthens our methodology because it reduces bias, making it possible to study the true prevalence of drug driving. Ethical and privacy challenges have been addressed to ensure that this study is compliant with the Tri-Council policy statement (TCPS) on ethical conduct for human research. The TCPS is the human research ethics policy used by Canadian research ethics boards. It promotes high ethical standards of conduct in research involving humans and is rooted in principles of respect for persons, concern for welfare and justice. The TCPS (Article 3.7A) allows waiver of consent in certain circumstances [35]. Key considerations are that: (a) research involves no more than minimal risk to the participants; (b) alteration to consent requirements is unlikely to adversely affect the welfare of participants; and (c) it is impossible or impracticable to carry out the research and to address the research question properly, given the research design, if the prior consent of participants is required. The REBs at all participating hospitals agree that the study meets TCPS requirements for waiver of consent. The study is considered low risk because it does not involve a procedure and has strict measures in place to prevent toxicology results from being linked to any individual driver.

### Chart review

The ED census is screened regularly to identify eligible drivers before their blood is discarded. Screening frequency depends on how quickly the hospital laboratory discards clinical blood samples. ED medical records of eligible drivers are reviewed, and relevant data is abstracted and entered in REDCap, a secure web application for building and managing online surveys and databases. ED records include ambulance records, emergency physician notes, nursing notes, laboratory results of blood alcohol concentration (BAC), and consultant notes (if applicable). The abstracted data includes age, sex, first three digits of postal code, arrival mode (via ambulance or walk-in), crash date and time, crash type (single or multiple vehicle crash), vehicle type, prescription medications used in last 30 days, medical history, documentation of alcohol or drug use, disposition and medications given as part of clinical care prior to blood draw (we account for “post-crash” medications when reporting toxicology results). We also determine the time interval from crash till blood is obtained based on ambulance record or nursing notes regarding time of crash and phlebotomists record of blood draw time.

### Blood handling and de-identification

After eligible drivers are identified, excess blood is obtained from the hospital laboratory and relabelled with study identification numbers (IDs replacing clinical labels. Blood samples are frozen at each site before shipment on dry ice by overnight courier to the central laboratory where samples are stored at − 40 °C until ready for toxicology analysis. Freezing is important as significant losses of many drugs will occur by 2 months if blood is stored at room temperature [36, 37]. Patient identifiers and study IDs are temporarily stored locally at individual hospital sites. Once clinical data for chart review is complete and data entry is verified by the coordinating site, the link between study IDs and patient identifiers is permanently destroyed. Toxicology analysis on samples is performed only after the link with patient identifiers is destroyed. Blood samples are relabelled a second time with a “toxicology ID” prior to toxicology analysis. A permanent link file between study ID and toxicology ID is maintained but the toxicology lab does not have access to study ID numbers. (Fig. 2).

### Toxicology analysis

Toxicology analyses are performed at British Columbia Provincial Toxicology Centre (PTC) using methods consistent with the 2007 Talloires report, *Guidelines for Drugged Driving Research* [25]. In participating hospitals, injured drivers are usually tested for alcohol as part of routine trauma care. If clinical alcohol levels are not available, alcohol is measured at the PTC using Gas Chromatography-Flame Ionization Detection with a detection limit of 0.01%. In addition, broad spectrum drug panels are performed on each patient’s blood using high-throughput liquid chromatography/tandem mass spectrometry (LC-MS/MS). The LC-MS/MS panel provides a quantitative measure of drug concentration using ISO-certified reference calibrators. The extraction process recovers both acidic and basic drugs and their metabolites including cannabinoids, cocaine, amphetamines and their major analogues, opioids, and impairing medications (Table 1). The method has detection limits of 0.2 ng/mL for THC and 1 ng/mL for most other substances. Our preferred sample is whole blood. When plasma is available but whole blood is not, we adjust plasma toxicology results to equivalent whole blood results according to previously published studies [17, 38].

**Table 1:** In addition to alcohol, the following list of potentially impairing drugs and medications are tested and quantified during toxicology analysis of the national drug driving study

Drug Classification	Drugs or Medications
Amphetamine	Amphetamine, MDA, MDMA, Methamphetamine
Analgesic	Tramadol
Anticonvulsant	Carbamazepine, Lamotrigine, Phenytoin, Topiramate, Gabapentin
Antidepressant	Venlafaxine, Hydroxybupropion, Citalopram, Fluoxetine, Mirtazepine, Norsertraline, Paroxetine, Sertraline, Trazodone, Bupropion, Norcitalopram, Nortriptyline, O-Desmethylvenlafaxine
Antiemetic	Metoclopramide
Antihistamine	Cetirizine, Chlorpheniramine, Diphenhydramine Tripelennamine, Doxylamine
Antipsychotic	Hydroxyrisperidone, Loxapine, Olanzapine, Quetiapine, Risperidone, Zuclopenthixol, Chlorpromazine, Clozapine, Haloperidol, Ziprasidone
Antitussive	Dextromethorphan
Benzodiazepine	Alprazolam, 7-Aminoclonazepam, 7-Aminoflunitrazepam, 7-Aminonitrazepam, Diazepam, Lorazepam, Midazolam, Oxazepam, Temazepam, Chlordiazepoxide, Clonazepam, Flunitrazepam, Nitrazepam, Nordiazepam
Cocaine and metabolites	Cocaine, Benzoylecgonine, Cocaethylene
Muscle relaxant	Cyclobenzaprine, Methocarbamol
Non-benzo hypnotic	Zopiclone, Zolpidem
Opiate	6-Acetylmorphine, Codeine, Hydromorphone, Methadone, Morphine, Oxycodone, EDDP, Fentanyl, Buprenorphine, Hydrocodone, Norfentanyl
Sedative	Ketamine, PCP
Tricyclic antidepressant	Amitriptyline, Doxepin, Trimipramine, Clomipramine, Desipramine, Imipramine
Cannabinoid	THC, COOH-THC
Others	Acetaminophen, Warfarin, Naloxone

### Data analysis

We use descriptive statistics to report the proportion of injured drivers, disaggregated by sex and age range, who test positive for the following classes of psychotropic drugs: alcohol, cannabis (COOH-THC, THC), cocaine, amphetamines, opioids, benzodiazepines, antihistamines, antidepressants, and antipsychotics. For drugs or drug classes with per se limits, we also report the proportion of drivers with drug levels above relevant limits (e.g., THC > 2 ng/mL; THC > 5 ng/mL in Canada). As drug use is generally higher in patients with severe injuries, we report prevalence separately for drivers with severe injuries (require hospital admission) and less severe injuries (treated and released from the ED). We also report the prevalence of various combinations of polysubstance use (e.g.*,* alcohol and cannabis). Regional variation is described by reporting drug prevalence separately for each region of the country.

## Discussion

Our methodology overcomes many limitations of previous research. We measure drugs in blood, which for most drugs, correlates better with impairment than drug levels measured in saliva or urine. Rather than merely detecting presence or absence of drugs, our methods quantify alcohol, THC, COOH-THC and 83 other impairing drugs and medications (Table 1). Additional “newly emerging” substances can be added to the toxicology panel in response to new information. This is a marked improvement over most roadside surveys because we detect more substances and we report drug levels in blood, which allows us to comment on probable impairment. Additionally, we use blood obtained shortly after the crash, in most cases within 1.5 h, so our toxicology results closely approximate drug levels at time of crash, [9] simplifying interpretation of toxicology findings. The decision to obtain blood in this study is not based on suspicion of drug use. Blood obtained for the study is collected when clinically indicated for managing the patient’s injuries, based on crash mechanism and/or physical examination. Clinicians do not receive drug testing results from this study. This process eliminates the selection bias that would occur if drug testing were based on suspicion of drug use. Also, as this study has ethics approval for waiver of consent, we avoid the bias that would arise if drivers who used drugs were less likely to consent for testing, as might be the case in roadside surveys.

We acknowledge several limitations. Hospitals in small cities do not treat many injured drivers making this study infeasible due to prohibitively high screening costs per driver. For this reason, we run this study only in larger trauma centres. We acknowledge that the prevalence of alcohol and/or drug use and the types of drugs used may differ in rural regions. This limitation is mitigated by the fact that the regional trauma centres chosen for this study provide trauma care for people in the surrounding rural areas, and our methods allow us to identify rural drivers (using the first three digits of postal codes). Another limitation is that we do not capture uninjured drivers or drivers with minor injuries who may have injured another road user in the collision. These drivers are difficult to study as they do not have blood tests in hospital and are rarely tested for drugs under the current legal system. Studying this population would require real-time identification and consent for drug testing, a process that would be both very expensive and likely subject to non-response bias.

Our methods are an innovative way to objectively monitor the prevalence and patterns of drug driving in most regions of Canada. These methods can be adapted in other countries and the resulting data can be used to inform policy and programs designed to reduce the prevalence of drug driving and for international comparisons.

## Data Availability

Not Applicable (this is a study protocol).

## Abbreviations

BAC: Blood alcohol concentration
COOH-THC: 11-Nor-9-carboxy-tetrahydrocannabinol (inactive metabolite of THC)
ED: Emergency Department
ID: Identification number
LC-MS/MS: Liquid chromatography/tandem mass spectrometry
PTC: Provincial Toxicology Centre
REB: Research ethics board
TCPS: Tri-Council policy statement
THC: Δ-9-tetrahydrocannabinol (main psychogenic compound in cannabis)
WHO: World health Organization
